# Anxieties, age and motivation influence physical activity in patients with myeloproliferative neoplasms - a multicenter survey from the East German study group for hematology and oncology (OSHO #97)

**DOI:** 10.3389/fonc.2022.1056786

**Published:** 2023-01-04

**Authors:** Sabine Felser, Julia Rogahn, Philipp le Coutre, Haifa Kathrin Al-Ali, Susann Schulze, Lars-Olof Muegge, Julia Gruen, Jan Geissler, Veronika Kraze-Kliebhahn, Christian Junghanss

**Affiliations:** ^1^ Department of Internal Medicine, Clinic III – Hematology, Oncology and Palliative Care, Rostock University Medical Center, Rostock, Germany; ^2^ Department of Hematology, Oncology, and Cancer Immunology, Charité Campus Virchow-Klinikum, Charité, Universitätsmedizin Berlin, Berlin, Germany; ^3^ Krukenberg Cancer Center Halle, University Hospital Halle, Halle (Saale), Halle, Germany; ^4^ Department of Internal Medicine, Medical Clinic II, Carl-von-Basedow-Klinikum, Merseburg, Germany; ^5^ Department of Internal Medicine III, Heinrich Braun Hospital, Zwickau, Germany; ^6^ LeukaNET/Leukemia Online e.V., Riemerling, Germany; ^7^ MPN-Netzwerk e. V., Bonn, Germany

**Keywords:** anxieties, education, fatigue, fears, health-related quality of life (HrQoL), myeloproliferative neoplasms (MPN), physical activity, sports

## Abstract

**Background:**

Physical activity (PA) is a non-pharmacological approach to alleviate symptom burden and improve health-related quality of life (HrQoL) in cancer patients (pts). Whether pts with myeloproliferative neoplasms (MPN) PA behavior changes due to symptom burden and/or knowledge of the putative beneficial effects of PA has not yet been investigated.

**Methods:**

We performed a large questionnaire study in MPN pts. Self-reported PA behavior and potential influencing factors of 634 MPN pts were analyzed. Questionnaires were used to assess demographics, anxiety, severity of symptoms, HrQoL, current level of everyday and sports activities, and the level of information regarding the importance/possibilities of PA. According to their PA, the pts were assigned to the three groups: “inactive”, “non-targeted active”, and “sporty active” and compared with each other.

**Results:**

Key findings are that in 73% of the pts, the disease had an impact on PA, with 30% of pts reducing their PA. The prevalence of anxieties (e.g., occurrence of thrombosis and bleeding) regarding PA was 45%. Sporty active pts had a lower symptom burden and better HrQoL (*p* ≤ 0.001) compared to the other groups. Inactive pts were significantly older and had a higher body mass index than sporty active pts. Inactive and non-targeted active pts felt less informed about the importance/possibilities of PA (*p* = 0.002).

**Conclusion:**

Our results suggest that especially older and non-sporty MPN pts could benefit from motivational as well as disease-specific PA information. This study was registered at the German Registry of Clinical Trials, DRKS00023698.

## 1 Introduction

Patients (pts) with myeloproliferative neoplasms (MPN) suffer from a variety of disease- and therapy-related symptom burden. In addition to fatigue, the most common symptoms include concentration problems, bone pain, headache, dizziness, microcirculatory symptoms, itching, night sweet, depression, and anxiety (1–4). In advanced disease, splenomegaly is common and often associated with abdominal discomfort, loss of appetite, and leads to weight loss in about one-fifth of pts (1, 5). All of these symptoms have implications on physical performance, emotional well-being, and health-related quality of life (HrQoL), and lead to work productivity impairments (1, 6, 7). Thanks to advances in diagnostics and therapy, many MPN pts have an almost normal life expectancy (8, 9). Of note, myelofibrosis (MF) -primary or secondary- is often associated with a more severe disease course and decreased overall survival (10). Pts with chronic myeloid leukemia (CML) benefit in regards to life expectancy from the effectiveness of tyrosinkinase inhibitors (TKI). Due to the predominantly chronic courses of the diseases, MPN pts suffer from symptoms throughout their lives. Thus, HrQoL is increasingly becoming a focus of MPN treatment.

Based on the evidence regarding the effects of physical activity (PA) on functionality, symptom burden, and HrQoL in pts with solid tumors, acute leukemia, lymphomas, and myelomas (11, 12), it is reasonable to assume that PA may be an effective non-pharmacological approach to reduce symptom burden and improve HrQoL in MPN pts (13). Whether MPN pts’ PA behavior changes due to symptom burden and/or knowledge of the putative beneficial effects of PA has not yet been investigated. Similarly, it is unclear whether the consequences of impaired hematopoietic system function have an impact on PA. MPN pts often have an increased risk of thrombosis and infection, an increased bleeding tendency and/or anemia, accompanied by a reduced performance capacity (3, 4, 9). Itching and skin reactions could also have an impact on PA.

To support MPN pts in maintaining or implementing a physically active lifestyle in the long term, targeted information is warranted. The present study investigated which factors show an association with PA in MPN pts. The present study investigated (I) whether and how PA behavior changes due to a MPN disease, (II) whether anxiety of certain events such as thrombosis, bleeding, and skin reactions have an influence on PA, (III) how physically inactive MPN pts differ from active pts, and (IV) whether MPN pts have knowledge of the importance and possibilities of PA.

## 2 Materials and methods

The study was designed as a multicenter cross-sectional study. It was approved by the Ethics Committee of the University of Rostock (A2020-0274) and registered with the German Registry of Clinical Trials: DRKS00023698. Pts ≥ 18 years with any type of MPN (14) could participate in the survey. Eligible pts of 12 institutions in the East German Study Group Hematology and Oncology (OSHO, *Online* **Supplementary S1**) were asked to participate and fill in a hard copy questionnaire (enrollment January 2021 to September 2021). From April 2021 to September 2021, the study was amended by an online version of the survey consisting of the same set of questions. Participants included pts of the LeukaNET/Leukemia-Online pts network as well as the German, Austrian, and Swiss MPN pts network.

### Questionnaire

*General information*. The following general characteristics were collected: gender, age, education level, family status, profession, height, and weight. The Body Mass Index (BMI) was calculated.

*Details of the disease*. MPN subtype, year of diagnosis, disease-specific therapies (e.g. TKI, januskinase (JAK) inhibitors, cytostatics (e.g. hydroxyurea, anagrelid, busulfan, clardibrin), inteferon, and other therapies (e.g., anticoagulation, phlebotomy) were inquired.

*Physical activity*. Questions asked for whether and how the PA had changed in everyday life and during sports since the diagnosis of the MPN, and whether they are afraid of certain events (e.g., bleeding). Everyday activity was measured with the Godin-Shepard Leisure-Time Physical Activity Questionnaire (GSLTPAQ) and was classified into three categories: “insufficiently active”, “moderately active”, and “active” (15, 16). Additionally, the five stages of the transtheoretical model of behavioral change (SOC) were used to determine the motivation to participate regularly in sports (17, 18). In the stages of precontemplation, contemplation, and preparation, pts are not regularly active in sports. In the stages of action and maintenance, pts are active for at least 20 minutes on at least 3 days per week. The questionnaires (GSLTPAQ, SOC) are provided in the *Online* **Supplementary S2**.

*HrQoL and symptoms.* HrQoL was assessed by a visual analogue scale (VAS) ranging from 0 (very poor) to 100 (very good). Symptoms were assessed using single items of the MPN Symptom Assessment Form (MPN-SAF) (19), supplemented by other typical symptoms of CML, ranging from 0 (absent) to 100 (worst imaginable). Further, weight changes, potential side effects of MPN such as skin reactions, splenomegaly, as well as the number of falls in the last 12 months were inquired.

*Information level.* It was recorded whether the pts felt sufficiently informed about the importance and possibilities of PA and the desire for more information.

### Activity groups

Pts were divided into three groups depending on their level of everyday (GSLTPAQ) and sports activities (SOC). Group 1 “inactive”: all insufficiently active pts who do no sports at all. Group 2 “non-target active”: all moderately and sufficiently active pts who do no sports at all. Group 3 “sporty active”: all moderately and sufficiently active pts who do sports regularly.

### Statistical analysis

Continuous data are reported as means ± standard deviation, and categorical variables as counts and percentages. Mean differences for continuous variables were tested using Mann-Whitney U test and χ^2^-test for categorical variables. All data were analyzed using SPSS (version 25.0, IBM, Chicago, IL, USA). Statistical significance was assumed for *p*-values < 0.05.

## 3 Results

### Sample characteristics

**Figure 1:**
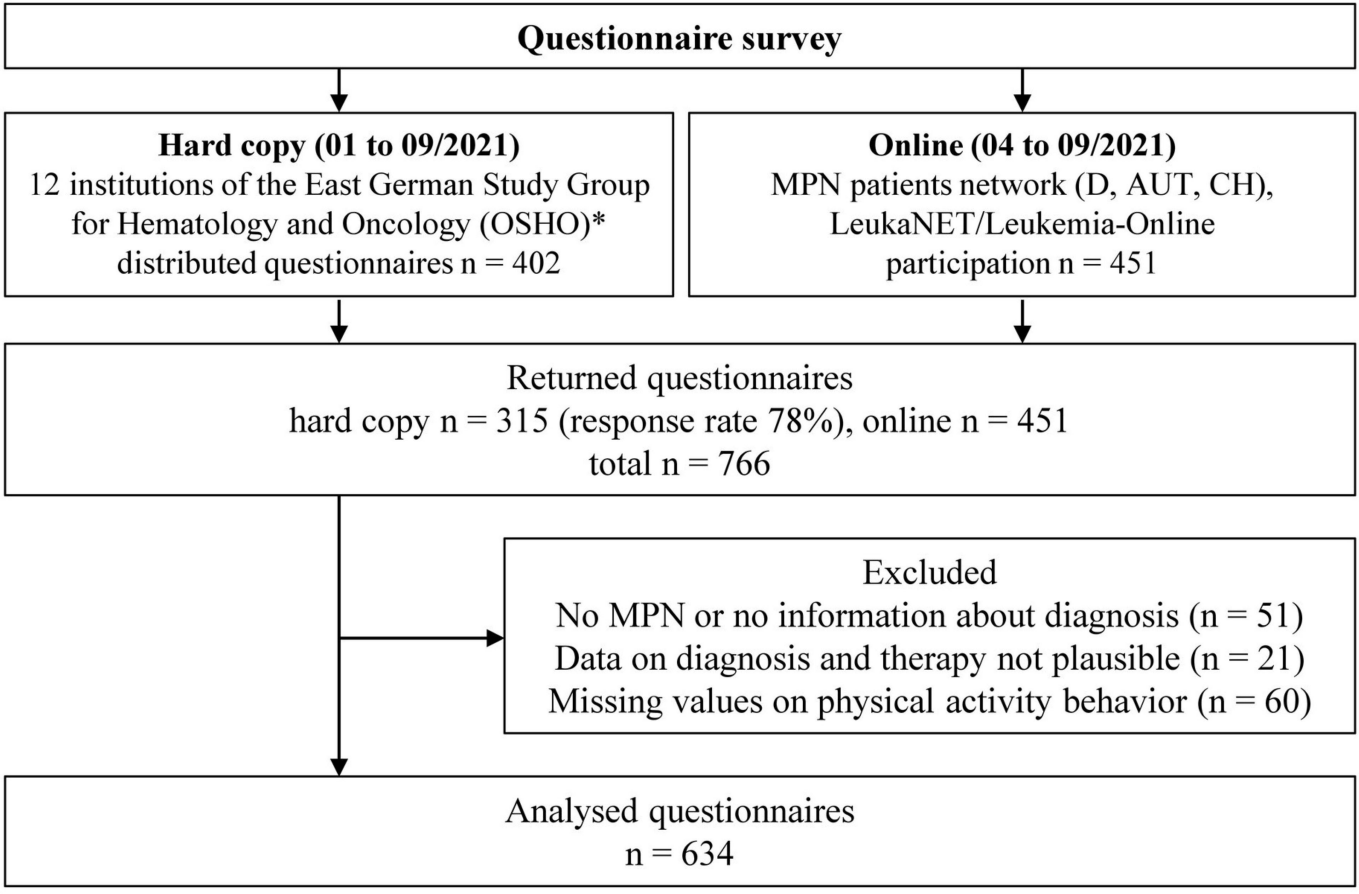
Flow chart of the study. *D*, Germany; *AUT*, Austria; *CH*, Switzerland; *MPN*, myeloproliferative neoplasms. *Participating institutions are presented in the supplement (**Table S1**).

In total, 766 questionnaires were received, of which 315 (41%) were in hard copy and 451 (59%) online. The response rate (handed out/received filled in) of the hard copy survey was 78%. Reasons for exclusion of questionnaires are presented in **Figure 1**. The final sample cohort comprised 634 questionnaires (63% women, mean age 57 ± 14 years). General characteristics, including the medical history of this cohort, are presented in **Table 1**. The pts were diagnosed between 1981 and 2021. The median age of MPN onset was 50 ± 14 years.

**Table 1:** Sample characteristics (n = 634).

	n	Values
General characteristics		
Gender	633	
women		398 (62.9)
men		235 (37.1)
Age [years]	632	57.1 ± 13.9
BMI [kg/m²]	627	25.8 ± 4.9
School education	609	
≤ 10 years		265 (43.5)
> 10 years		344 (56.5)
Family status	630	
single		107 (17.0)
married/living with a partner		483 (76.7)
other		40 (6.3)
Profession	624	
working*		339 (54.3)
retired		239 (38.3)
other		46 (7.4)

Medical history		
Year of diagnosis	586	
≥ 2020		90 (15.4)
≥ 2010 and < 2020		371 (63.3)
≥ 2000 and < 2010		108 (18.4)
< 2000		17 (2.9)
MPN subtype	634	
CML		183 (28.9)
PV		166 (26.2)
ET		155 (24.4)
MF		117 (18.5)
others		13 (2.1)

Data are presented as the number of participants (%) for categorical variables and as mean ± standard deviation for continuous variables.

n, number of patients; BMI, Body Mass Index; CML, chronic myeloid leukemia; PV, polycythemia vera; ET, essential thrombocythemia; MF, myelofibrosis

*42 (12.1%) patients were on sick leave at the time of the survey.

Further demographics and current therapies at the time of the survey are presented in **Table 2**. The CML pts were the youngest pts (mean 51 ± 14 years), the polycythemia vera (PV) pts were the oldest (mean 61 ± 12 years). Of the 183 CML pts, 171 received disease-specific therapy. Of these, 158 (92%) were treated with TKI. One hundred thirty-two of the 166 PV and 133 of the 155 essential thrombocythemia (ET) pts received disease-specific therapy, most frequently with cytostatics (n = 55, 42% and n = 70, 53%, respectively). Thirteen (10%) of PV and 36 (27%) of ET pts were on a watch-and-wait strategy. The most common disease-specific therapy among MF pts was treatment with a JAK-2 inhibitor, (n = 56, 52%) followed by a watch-and-wait strategy (n = 22, 20%). Regardless of disease-specific therapy, 66 (40%) PV, 87 (56%) ET, and 31 (26%) MF pts received anticoagulation. Sixty-three (38%) PV pts underwent phlebotomy. The total cohort included seven pts with splenectomy.

**Table 2:** Demographics and current therapies of patients with myeloproliferative neoplasms depending on the diagnosis.

	CML	PV	ET	MF
Total sample size	n = 183	n = 166	n = 155	n = 117
Demographics				
Gender, women	100 (54.6)	113 (68.1)	113 (72.9)	67 (57.3)
Age [years]	51.1 ± 13.6	61.0 ± 12.2	56.7 ± 15.3	59.9 ± 11.1
BMI [kg/m²]	26.4 ± 5.3	25.2 ± 4.7	25.2 ± 4.2	26.3 ± 5.3
School education, ≤ 10 years	69 (37.7)	71 (42.8)	70 (45.2)	49 (41.9)
Profession, retired	43 (23.5)	75 (45.2)	62 (40.0)	50 (42.7)
Disease-specific therapies	n = 171	n = 132	n = 133	n = 108
Tyrosine kinase inhibitor	158 (92.4)	–	–	–
Januskinase inhibitors	–	38 (28.8)	10 (7.5)	56 (51.9)
Cytostatics	7 (4.1)	55 (41.7)	70 (52.6)	19 (17.6)
Interferon	6 (3.5)	35 (26.5)	20 (15.0)	15 (13.9)
Stem cell transplantation	7 (4.1)	–	–	4 (3.7)
Watch-and-wait	–	13 (9.8)	36 (27.1)	22 (20.4)

Data are presented as the number of participants (%) for categorical variables and as mean ± standard deviation for continuous variables.

CML, chronic myeloid leukemia; PV, polycythemia vera; ET, essential thrombocythemia; MF, myelofibrosis; BMI, Body Mass Index

### Influence of MPN disease on physical activity and anxiety

**Figure 2:**
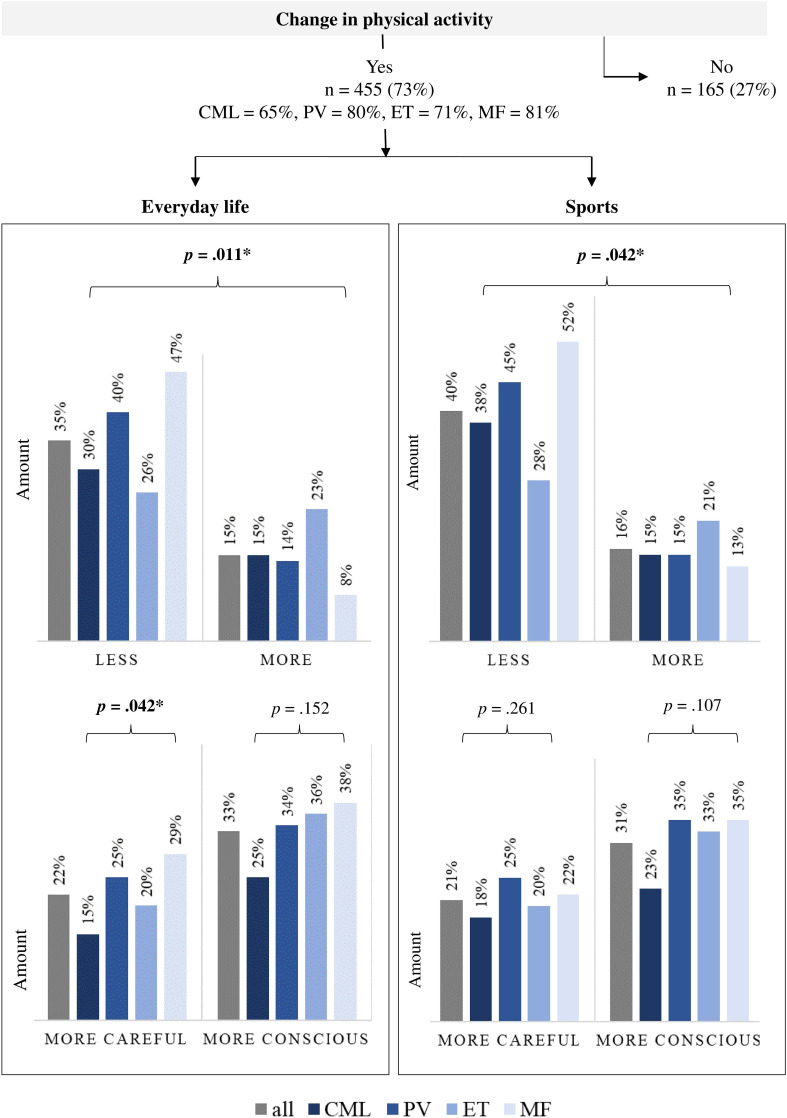
Influence of a myeloproliferative neoplasia disease on self-reported physical activity (n = 620). n, number of patients; CML, chronic myeloid leukemia (n = 169); PV, polycythemia vera (n = 166); ET, essential thrombocythemia (n = 155); MF, myelofibrosis (n = 117). Bold: statistically significance, *p ≤.05.

The influence of a MPN disease on the PA of those affected is presented in **Figure 2**. Most participants (n = 455, 73%) changed their self-reported PA behavior in everyday life and/or sports. Both, the decade of diagnosis and the type of therapy in PV/ET/MF pts showed no significant group differences. Group differences were only found depending on the diagnosis. The percentage of those who changed their PA behavior was lowest among the CML pts (65%) and highest among the MF pts (81%). In everyday life, 177 (35%) reported being less active and 78 (15%) more active. Especially pts with MF and PV reduced their everyday activities (47% and 40% respectively). The proportion of pts who have been more active in everyday life since diagnosis is highest among ET pts (23%) and lowest among MF pts (8%). Two hundred one pts (33%) moved more consciously in everyday life and 132 (22%) moved more carefully. The latter finding was especially relevant in pts with MF and PV (29% and 25%, respectively). In sports, 191 (40%) of the 455 pts reduced their training, with the proportion being lowest among ET pts at 28% and highest among MF pts at 52%. In contrast, 76 (16%) reported exercising more since diagnosis, which was most common for ET pts (21%). One hundred and eighty-one (31%) were more conscious during sports and 123 (21%) were more careful.

**Figure 3:**
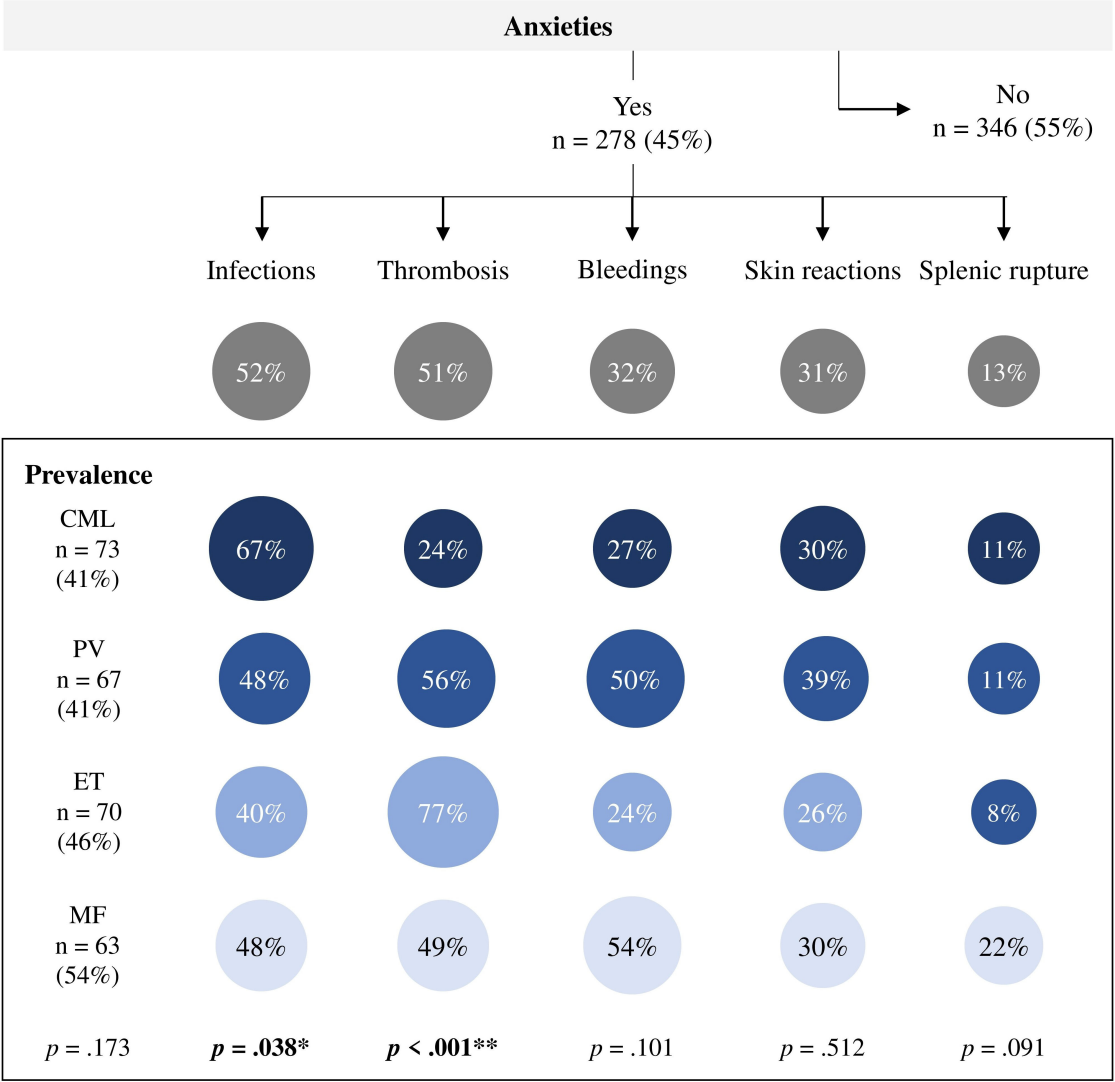
Anxieties during physical activity in patients with myeloproliferative neoplasms (n = 624). n, number of patients; CML, chronic myeloid leukemia (n = 178); PV, polycythemia vera (n = 164); ET, essential thrombocythemia (n = 155); MF, myelofibrosis (n = 117). bold: statistically significance, *p ≤ 05, **p ≤ 001.

Thirty percent of those who moved/exercised more consciously were more physically active. In contrast, 68% of those who were more careful were less physically active. Pts who reported moving less after diagnosis tended to have more anxiety about certain events than pts who reported moving as much or more (54% vs. 31% and 33%, respectively, *p* ≤ 0.001). Which events MPN pts are most afraid of during PA are presented in **Figure 3**. Overall, 278 (45%) participants reported that they were afraid of at least one event. There were no significant group differences in the prevalence of anxiety according to diagnosis. Anxiety about infections (52%) and thrombosis (51%) were mentioned most frequently, followed by bleeding (32%) and skin reactions (31%). Group differences were found in anxiety about infections (*p* = 0.038) and thrombosis (*p* ≤ 0.001). While CML pts had more anxiety about infections, ET and PV pts had more anxiety about thrombotic events compared to the other MPN subtypes. MF pts tended to have more anxiety about splenic rupture compared to the other MPN subtypes (22% *vs*. 8-11%, respectively, *p* = 0.091).

### Physical activity level and motivation for regular sports

Regarding the GSLTPAQ score, 110 (19%) of the total cohort were categorized as insufficiently active, 109 (19%) as moderately active, and 361 (62%) as active. The analysis of SOC revealed that 203 (34%) pts were not action-oriented (stage of precontemplation), 98 (16%) and 41 (7%) were in the stages of contemplation and preparation, respectively. In total, 257 (43%) pts reported regular sports (stages of action and maintenance). The results for the different diagnoses are available in the *Online* **Supplementary S3**. There are no significant group differences in everyday activity (GSLTPAQ) or motivation to do regular sports (SOC) depending on diagnosis or therapy in PV/ET/MF pts.

### Activity groups

**Figure 4:**
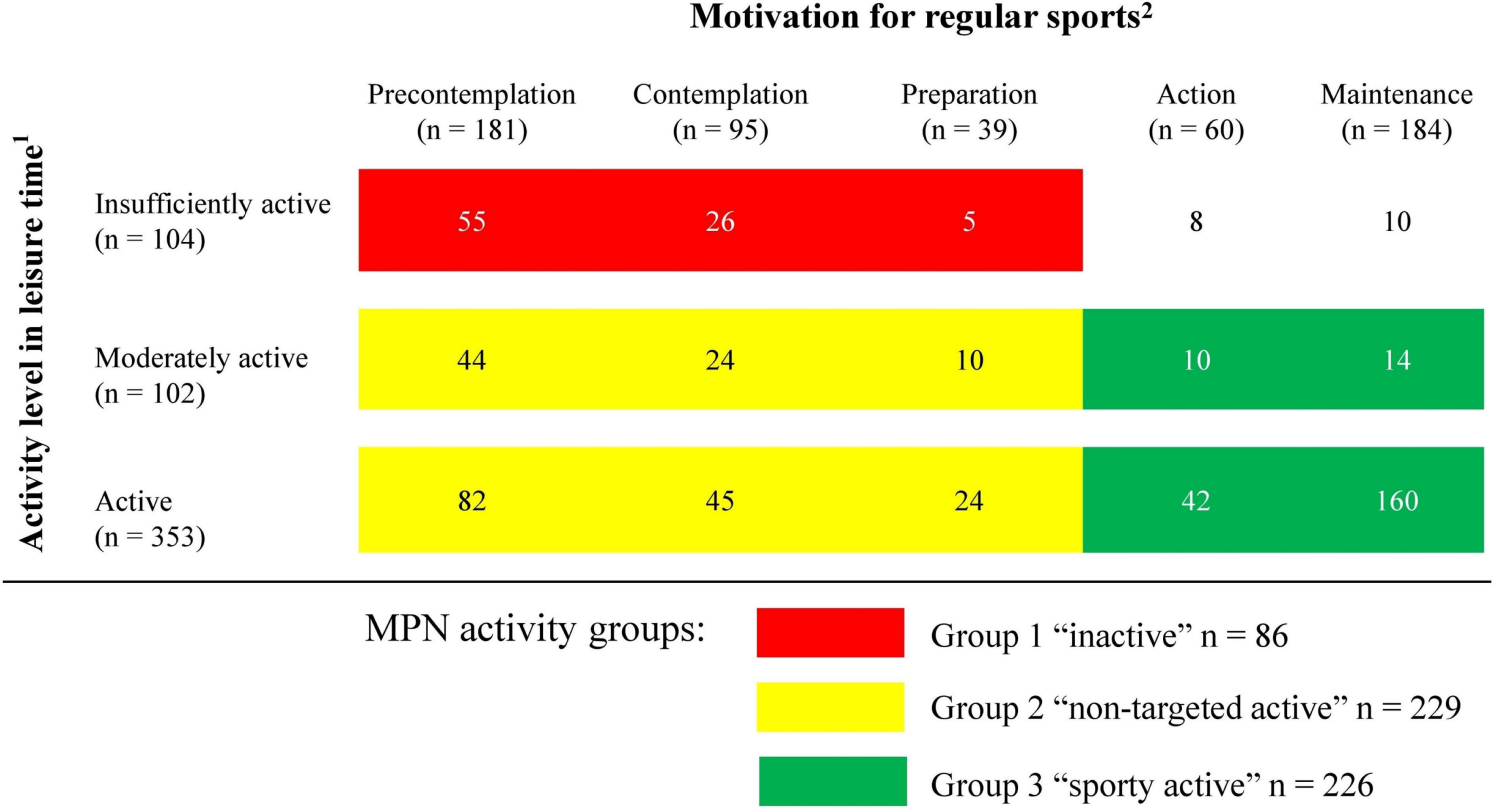
Physical activity in patients with myeloproliferative neoplasms (n = 559). Data are presented as the number of participants. ^1^Activity levels in leisure time were grouped according to the Godin-Shepard Leisure-Time Physical Activity Questionnaire (GSLTPAQ) into three categories: “insufficiently active”, “moderately active”, and “active” (15, 16). ^2^Five stages of the transtheoretical model of behavioral change (SOC) were used to determine the motivation to participate in sports. In the stages of precontemplation, contemplation, or preparation, patients are not regularly active in sports. In the stages of action and maintenance, patients are active for at least 20 minutes on at least 3 days per week (17, 18).

All MPN pts who reported both GSLTPAQ and SOC (n = 559) were assigned to an activity group according to the information provided. For 18 (3%) pts, the information provided was not plausible. These were excluded from the subgroup analysis. The SOC as a relation to the GSLTPAQ scores are presented in **Figure 4**. Eighty-six (15%) pts were assigned to Group 1 “inactive”, 229 (41%) to Group 2 “non-targeted active”, and 226 (40%) to Group 3 “sporty active”.

### Demographics, HrQoL, and symptom burden depending on the activity group

The demographics, HrQoL, symptoms, and side effects of the MPN pts, depending on the activity group, are presented in **Table 3**. The inactive pts were significantly older than those in the two active groups (Group 2: 60 ± 16 years vs. 56 ± 13 years; *p* = 0.018; Group 3: 55 ± 13 years; *p* = 0.004), and had a higher BMI than the sporty active group (27 ± 5 vs. 25 ± 5, *p* = 0.013). The sporty active group rated their HrQoL significantly higher than the other two groups (Group 1: 73 ± 20 vs. 60 ± 23, *p* ≤ 0.001 vs. Group 2: 63 ± 21, *p* ≤ 0.001). In addition, the sporty active group reported significantly less fatigue, bone and muscle pain, and concentration problems (all: *p* ≤ 0.05), compared to both groups. There were no differences in HrQoL and symptom burden between the inactive and non-targeted active pts.

**Table 3:** Characteristics of patients with myeloproliferative neoplasms depending on the level of physical activity.

	Inactive (1) (n = 86)	non-targeted active (2) (n = 229)	sporty active (3) (n = 226)	*p*-value group 1 vs 2	*p*-value group 2 vs 3	*p*-value group 1 vs 3	*p*-value χ^2^-test
Demographics							
Gender, women	64.0	66.7	58.8				.224
Age [years]	59.5 ± 15.5	56.0 ± 12.9	54.7 ± 12.9	.018*	.363	.004*	
BMI [kg/m²]	26.6 ± 5.2	25.8 ± 4.4	25.3 ± 4.8	.319	.072	.013*	
School education, ≤ 10 years	47.6	43.2	32.3				.095
Profession, retired	42.9	36.9	31.0				.214
**Health-related quality of life^1^**	60.1 ± 22.7	63.2 ± 21.2	73.2 ± 19.7	.266	≤.001**	≤.001**	
Symptoms^2^							
Fatigue	45.6 ± 31.5	43.9 ± 29.8	33.0 ± 28.1	.656	≤.001**	≤.001**	
Bone and muscle pain	37.3 ± 31.9	33.1 ± 28.9	25.0 ± 27.6	.467	≤.001**	.005*	
Concentration problems	31.7 ± 28.6	35.0 ± 27.5	23.9 ± 25.5	.283	≤.001**	.024*	
Itching	16.7 ± 24.6	16.9 ± 24.2	13.3 ± 22.8	.430	.027*	.438	
Abdominal discomfort	20.6 ± 26.9	19.4 ± 26.6	15.7 ± 23.5	.605	.236	.156	
Early satiety	24.3 ± 27.0	18.6 ± 25.6	16.2 ± 23.3	.082	.394	.016*	
Night sweats	20.8 ± 24.7	21.2 ± 29.0	16.8 ± 26.7	.530	.204	.069	
Fever (> 37,8°C)	0.9 ± 3.4	1.2 ± 4.8	1.5 ± 6.7	.177	.560	.330	
**Weight change during last 3 mths**							.070
weight gain, yes	29.4	24.6	18.7				
unintended weight loss, yes	9.4	9.2	8.4				
intended weight loss, yes	0.0	8.3	8.4				
Current side effects/concomitants							
Skin reactions, yes	41.9	48.0	40.3				.267
Splenomegaly, yes	32.6	25.8	31.0				.026*
increased bleeding tendency, yes	22.1	33.3	31.4				.242
Thrombosis during last 3 month, yes	7.0	1.7	4.9				.038*
**Falls during last 12 months, yes**	23.3	13.1	9.7				.007*
Information on physical activity							
felt sufficiently informed, yes	47.1	48.7	63.6				.002*
more information desired, yes	68.6	67.1	63.6				.603

Data are presented as mean ± standard deviation for continuous variables and as percentage of patients for categorical variables.

Mean differences for continuous variables were tested using Mann-Whitney U test and χ^2^-test for categorical variables.

n, number of patients; BMI, Body Mass Index.

^1^range 0-100, higher values represent high health-related quality of life; ^2^range 0-100, higher values represent more discomfort.

bold: statistically significance, *p ≤.05, **p ≤.001.

Inactive MPN pts tended to gain weight more often than the active ones (29% vs. 25% and 19%, respectively, *p* = 0.070). Eight percent of the active pts intentionally lost weight.

The inactive pts reported thromboses more often than the active pts (7% vs. 2% and 5%, respectively, *p* = 0.038). Falls during the last 12 months were reported significantly more often in this group, as well (23% vs. 13% and 10%, respectively, *p* = 0.007). Thirty-one percent of the sporty active group reported a splenomegaly, which was in the range to the inactive (33%, *p* = ns), but of note: in higher proportion compared to the non-targeted active (26%, *p* = 0.026).

### Association between information level and physical activity

Two hundred and seventy-two (43%) of all participants stated that they did not feel sufficiently informed about the importance and possibilities of PA for their disease. The differences in the level of information depending on the activity group are presented in **Table 3**. Uninformed pts belonged significantly more often to the group of inactive and non-targeted active pts (*p* = 0.002). All pts, regardless of their activity level, expressed their wish to receive more information about PA.

## 4 Discussion

This is the first study to investigate the PA behavior of MPN pts and provide an overview of which factors show an association with PA in this population. The most important results are discussed below and information is derived for which pts may need to lead an active lifestyle for as long as possible.

According to the presented data, the MPN disease and associated therapies had an impact on PA in 65-81% of the pts, depending on the MPN subtype. Approximately one in three pts reported a reduction in PA because of the disease, with the proportion highest in MF pts and lowest in ET pts. This is about as expected, as symptom burden varies according to diagnosis and consequently has different effects on HrQoL. Furthermore, the prevalence of moderate to severe fatigue, which is associated with a reduction in PA, is about 50% in MPN pts (1, 11).

Of importance is the result that in many MPN pts, depending on the MPN subtype, fear of certain events - especially infections, thromboses, bleeding, and skin reactions - had a negative influence on PA behavior. The reduction was not only limited to sports activities, but also affected everyday activities. Of particular interest is our finding that PV and ET pts more frequently reported anxieties compared to CML and MF pts in regards to the occurrence of thromboses. This might reflect the fact, that in PV and ET thromboembolism is often the initial disease complication that leads to the diagnosis. The rare occurrence of thrombosis in the present cohort (3%) suggests that the fear especially of PV and ET pts has to be addressed in order to avoid negative effects on PA. The high proportion of pts in the present study who regularly participate in sports despite anticoagulation and/or skin reaction suggests that these symptoms do not represent a limitation to sports activities. The numerous exercise interventions available for pts following high-dose chemotherapy and hematopoietic stem cell transplantation also suggests that exercise is safe for pts at increased risk of bleeding and infection, and that pts may benefit from numerous positive effects (20). It cannot be excluded that the COVID-19 pandemic prevailing at the time of the survey increased the fear of infection, which was not further specified. The present results suggest that MPN pts should be informed about the real risk of thrombosis or serious bleeding during PA. To support patients’ active lifestyles, ways to reduce the risk of infection, bleeding, and/or skin reactions during daily activities and sports should be emphasized (e.g., hand disinfection, face mask during group exercise, low-injury sports/forms of exercise, and if necessary, refrain from water sports, add sun protection, etc.).

According to the presented data, 62% of MPN pts were sufficiently physically active in their daily lives at the time of the survey (self-reported), and 43% stated that they regularly played sports. These are unexpectedly high percentages and might be due to the survey design. Due to the voluntariness, it cannot be ruled out that more pts with an affinity for sports took part in the survey. In addition, 10% of the participants did not answer the questions on PA behavior or answered them inadequately and were excluded from the analysis. Furthermore, the cohort is quite young with an average age of 57 years and it is known that PA tends to decrease with age (21). Likewise, socially desirable responses cannot be excluded. Based on the results of large American and British cohort studies of cancer survivors, it must be assumed that the proportion of insufficiently physically active MPN pts is higher than the results of this study show (22, 23). Regardless, the participants could be divided into three groups (inactive, non-targeted active, and sporty active) according to their PA statements, which were sufficiently large for the statistical analyses.

Among physically active pts, the proportion of pts with increased bleeding tendency or skin reactions is as high as among inactive pts. Consequently, these side effects/concomitants are not, or only to a limited extent, barriers to PA or sports. Mean comparisons showed comparable symptom burden and HrQoL for the inactive and non-targeted active pts. This suggests that a lack of motivation is the reason for inactivity rather than symptom burden. As behaviors tend to become entrenched over time, they are often difficult to change. To motivate previously inactive MPN pts to adopt an active lifestyle, a psychologist’s involvement may be beneficial. The most effective strategies to motivate cancer pts to be more physically active in the long term include motivational interviewing, coaching, and Bandura’s socio-cognitive learning (model learning) approach (24).

In all groups, fatigue, bone and muscle pain, and concentration problems represented the most common severe symptoms. The reported prevalence and severity of these symptoms is comparable to results of other studies (1, 11). Inactive and non-targeted active pts showed no differences in symptom burden and HrQoL. Based on the findings that PA and fatigue correlate negatively, and PA and HrQoL correlate positively (5, 11), this is an unexpected result. However, the result could be an indication that it is not the amount of PA that is decisive for the symptom burden, but the quality/targeting of the PA. This is also in line with the general recommendations for reducing fatigue. Moderate-intensity exercise is recommended here, as the effect is unlikely at low intensities. Moreover, there is no evidence for a dose-response relationship (25).

The lower symptom burden and higher HrQoL of the sporty active pts in our study is consistent with the assumption of Eckert et al. (13) that targeted PA could also have positive effects in MPN pts. However, as there is a bidirectional relationship between symptom burden or HrQoL and PA, no statement on causality can be made on the basis of the available cross-sectional data. This should be investigated in subsequent studies. Due to the small differences in symptom burden and HrQoL between the groups (about 10%), it is assumed that the effects of targeted PA on symptoms and HrQoL in MPN pts are modest. This is also confirmed by the results of Huberty et al. (26, 27). Thus, small to moderate effect size for sleep disturbance, pain intensity, anxiety, and depression were generated by approximately one hour of yoga training per week over a period of 12 weeks. Although Pedersen et al. (28) demonstrated that a 12-week self-exercising program, after a 5-day interdisciplinary exercise-based rehabilitation intervention, significantly increased physical performance of MPN pts, but no improvements were seen with respect to HrQoL and fatigue. However, since PA has a multitude of health potentials, MPN pts should be motivated to be physically active regularly and for as long as possible. The available data suggest that both daily activities and sports can reduce the risk of falls and regulate body weight. The fact that 12% of pts surveyed reported being more conscious and/or increasing their PA since diagnosis suggests that adequate patient education can alleviate potential fears and possibly increase motivation to engage in PA or sports. The focus should be specifically on older and physically inactive pts (29). Similarly, overweight MPN pts should be addressed, appropriately educated, and motivated to be physically active. Reducing obesity in MPN pts might have several positive effects on outcome. Reducing obesity associated diseases such as atherosclerosis and risk factors is a general goal. In particular as some MPN treatment approaches such as TKI-treatment or stem cell transplantation might increase the risk for e.g. atherosclerosis themselves. Furthermore, obesity might influence pharmacokinetics of drugs used in MPN treatment, although data is limited (30–33).

The presented data is based on a large cohort, but due to the survey design, there are inevitable limitations that should be taken into consideration when interpreting the data. First, due to the online format, no information is available on how many potential participants were informed about the study and declined to participate. Due to the high proportion of online questionnaires, a bias towards more women and “younger” respondents is suspected. Even though it is known that women often report a higher symptom burden, we suspect that the bias of the results due to the unbalanced participation of the genders is small (34). This assumption is strengthened by the fact that the proportion of women in the three activity groups did not differ significantly. Second, all data were assessed retrospectively. Third, due to the cross-sectional design of the study, it is not possible to distinguish between cause and effect in terms of symptom burden and PA. Regardless of whether and to what extent individual symptoms can be reduced by PA, our results highlight the importance of PA because of its multitude of other potentials, such as reducing the risk of falls and weight control. Forth, in order to reduce the length of the questionnaire, no validated questionnaire was used for the assessment of HrQoL, but a VAS scale from 0 to 100. Since the VAS allows a more differentiated assessment of HrQoL compared to a Likert scale, it can be assumed that the HrQoL of cancer pts can be measured just as adequately (35). A major advantage of our study is the relatively large sample size. This representative sample of the population-based study thus enables the transfer of the results to clinical practice.

## 5 Conclusion

In conclusion, it could be shown that the majority of MPN pts change their self-reported PA behavior due to the MPN disease or therapy. About one third of all MPN pts reduce the amount of PA, especially pts with PV and MF. In addition to fears, especially of infection, thrombosis and bleedings depending on the MPN subtype, higher age and motivation level also seem to influence PA. Sporty pts have a lower symptom burden and higher HrQoL than non-sporty pts. Physically inactive pts have a significantly higher prevalence of falls and higher BMI compared to physically active pts. Inactive and non-targeted active pts were significantly less likely to be informed about the importance and possibilities of PA. Our data clearly suggest that PA information and education, as well as sports programs, should be integrated into the treatment of MPN pts. Further studies, especially longitudinal studies are needed to verify the results of the survey study.

## Data availability statement

The raw data supporting the conclusions of this article will be made available by the authors, without undue reservation.

## Ethics statement

The studies involving human participants were reviewed and approved by Ethics Committee of the University of Rostock. Written informed consent for participation was not required for this study in accordance with the national legislation and the institutional requirements.

## Author contributions

Conception and design: SF, CJ. Statistical analysis and interpretation: SF, JR, CJ. Data collection: PC, HA-A, SS, L-OM, JuG, JaG, VK-K. Writing the article: SF, JR, CJ. Critical revision of the article: SF, JR, PC, HA-A, SS, L-OM, JuG, JaG, VK-K, CJ. Obtained funding: SF. Overall responsibility: SF, CJ. All authors contributed to the article and approved the submitted version.
