# Supplementary material for: Anxieties, age and motivation influence physical activity in patients with myeloproliferative neoplasms - a multicenter survey from the East German study group for hematology and oncology (OSHO #97)
Source: Front Oncol. 2023 Jan 4;12:1056786. doi: 10.3389/fonc.2022.1056786 (PMC9846799; doi:10.3389/fonc.2022.1056786)
Supplement: Supplementary file 1 [file DataSheet_1.docx]

S1. Member institutions of the East German Hematology and Oncology Study Group (OSHO) participating in the survey

| **Number** | **Institution** |
| --- | --- |
| 1 | Department of Internal Medicine, Clinic III – Hematology, Oncology and Palliative Care, Rostock University Medical Center |
| 2 | Department of Hematology, Oncology, and Cancer Immunology, Campus Virchow‐Klinikum, Charité, Universitätsmedizin Berlin |
| 3 | Krukenberg Cancer Center Halle, University Hospital Halle (Saale) |
| 4 | Department of Internal Medicine, Medical Clinic II - Hematology, Oncology, Palliative Medicine, Carl-von-Basedow-Klinikum, Merseburg |
| 5 | Department of Internal Medicine III, Heinrich Braun Klinikum Zwickau |
| 6 | Department of Oncology and Hematology, Asklepios Klinik Weissenfels |
| 7 | Oncology and Hematology Practice, Lübsche Straße 146, Wismar |
| 8 | Department of Haematology and Oncology, Leipzig University |
| 9 | Oncology and Hematology Practice, Wismarsche Str. 32, Rostock |
| 10 | Department of Hematology and Oncology, Paul Gerhardt Stift, Wittenberg |
| 11 | Department of Hematology and Oncology, Klinikum Südstadt Rostock |
| 12 | Oncology and Hematology Practice, Goethestraße 1A, Parchim |

S2A. The Godin-Shepard Leisure-Time Physical Activity Questionnaire

During a typical 7-day period (a week), how many times on the average do you do the following kinds of exercise for more than 15 minutes during your free time (write on each line the appropriate number)?

|  | Times per week |  | Totals |
| --- | --- | --- | --- |
| **STRENUOUS EXERCISE (HEART BEATS RAPIDLY)**  (e.g., running, jogging, hockey, football, soccer, squash, basketball, cross country skiing, judo, roller skating, vigorous swimming, vigorous long-distance bicycling) |  | X9 |  |
| **MODERATE EXERCISE (NOT EXHAUSTING)**  (e.g., fast walking, baseball, tennis, easy bicycling, volleyball, badminton, easy swimming, alpine skiing, popular and folk dancing) |  | X5 |  |
| **MILD/LIGHT EXERCISE (MINIMAL EFFORT)**  (e.g., yoga, archery, fishing from river bank, bowling, horseshoes, golf, snow-mobiling, easy walking) |  | X3 |  |
| **WEEKLY LEISURE-TIME ACTIVITY SCORE** |  |  |  |

**Weekly leisure activity score** = (9 × Strenuous) + (5 × Moderate) + (3 × Light)

| **Godin Scale Score** | **Interpretation** |
| --- | --- |
| 24 units or more | Active |
| 14 – 23 units | Moderately Active |
| Less than 14 units | Insufficiently Active/Sedentary |

The GSLTPAQ was found to demonstrate good test-retest performance, with a correlation coefficient of 0.69 for the overall leisure score index and a range from 0.24 to 0.84 for the measurement of light, moderate, and strenuous activity. The correlation between leisure score index and maximum oxygen consumption was found to be 0.56.

**Reference:**

Godin, G. (2011). The Godin-Shephard leisure-time physical activity questionnaire. Health & Fitness Journal of Canada, 4(1), 18-22.

Jacobs DR, Jr., Ainsworth BE, Hartman TJ, Leon AS. A simultaneous evaluation of 10 commonly used physical activity questionnaires. Med Sci Sports Exerc. 1993;25(1):81-91.

S2B. Five stages of the transtheoretical model of behavioral change

1. *Intensive physical activity* refers to activities such as jogging, aerobics, speedy cycling, swimming which are performed to increase physical fitness and which usually make you sweat. One speaks of *regular physical activity* if this activity lasts in each case at least 20 minutes and is performed at least on 3 days per week.
2. Are you currently executing *intensive physical activity* on a regular basis for at least 3 days a week for 20 minutes each? Please mark which statement suits you best (please tick only one answer!)

□_1_ No, and I do not intend to start in the next 6 months.

□_2_ No, but I intend to start within the next 6 months.

□_3_ No, but I intend to start within the next 30 days.

□_4_ Yes, but only for less than 6 months.

□_5_ Yes, for more than 6 months.

1. Have you taken any steps in the last 6 months to become physically more active (e.g. bought sports equipment, inquired about an association, walked more)?

□_1_ No □_2_ Yes, what? _____________________________________________________________

Five stages of the transtheoretical Model of behavioral change (SOC) were used to determine the motivation of MPN patients to participate in sports. In order to assign the patients to the SOC, an algorithm was used describing regular sport. By answering the first question, patients were assigned to the stages of precontemplation (stage 1), contemplation (stage 2), action (stage 4) or maintenance (stage 5). Consideration of the second question allowed the assignment to the stage contemplation of preparation (stage 3), however, only if the patients had done something to prepare for physical training (e.g. bought sports equipment) within the last six months. If not, they were assigned to the stage of contemplation (stage 2).

**References:**

Prochaska JO, Redding CA, Evers KE. “The transtheoretical model and stages of change,”. In: Glanz K, Rimer BK, Viswanath K, editors. Health Behavior and Health education: Theory, Research and Practice. Hoboken, NJ: Jossey-Bass (2008). p. 97–122.

Prochaska JO, Marcus BH. “The transtheoretical model: Applications to exercise,”. In: Dishman RK, editor. Advances in exercise adherence. Champaign, IL, England: Human Kinetics Publishers (1994). p. 161–80.

S3. Physical activity level of patients with myeloproliferative neoplasms depending on the diagnosis

|  | CML | PV | ET | MF | *p*-value |
| --- | --- | --- | --- | --- | --- |
| Total sample size | n = 183 | n = 166 | n = 155 | n = 117 |  |
| **Activity level in leisure time^1^**  insufficiently active  moderately active  active | **169 (100)**  38 (22.5)  35 (20.7)  96 (56.8) | **149 (100)**  26 (17.4)  32 (21.5)  91 (61.1) | **139 (100)**  17 (12.2)  24 (17.3)  98 (70.5) | **111 (100)**  24 (21.6)  18 (16.2)  69 (62.2) | .184 |
| **Regularly active in sport^2^**  Precontemplation  no  Contemplation  Preparation  Action  yes  Maintenance | **170 (100)**  49 (28.8)  29 (17.1)  15 (8.8)  20 (11.8)  57 (33.5) | **157 (100)**  60 (38.2)  21 (13.4)  10 (6.4)  16 (10.2)  50 (31.8) | **147 (100)**  52 (35.4)  22 (15.0)  9 (6.1)  15 (10.2)  49 (33.3) | **115 (100)**  37 (32.2)  25 (21.7)  8 (7.0)  13 (11.3)  32 (27.8) | .817 |

Data are presented as the number of participants (%).

Abbreviations: *CML*; chronic myeloid leukemia, *PV*; polycythemia vera, *ET*; essential thrombocythemia, *MF*; myelofibrosis;

^1^The Godin-Shepard Leisure-Time Physical Activity Questionnaire was used to assess the level of physical activity of each participant. The average frequency of light, moderate and strenuous activity is recorded, i. e. more than 15 minutes each over the course of the last seven days. The "weekly activity score" is calculated for evaluation. The intensity categories are equal to: light = 3 MET (Metabolic Equivalent of Task); moderate = 5 MET; vigorous = 9 MET. Physical activity was classified into three categories: insufficiently active (Score < 14), moderately active (Score 14 to 23), active (Score ≥ 24).^15,16^

^2^Five stages of the transtheoretical model of behavioral change were used to determine the motivation to participate in sports. In the stages of precontemplation, contemplation or preparation, patients are not regularly active in sports. In the stages of action and maintenance patients are active for at least 20 minutes on at least 3 days per week.^17,18^
